# Synthesis and Antibacterial Evaluation of a Series of 11,12-Cyclic Carbonate Azithromycin-3-*O*-descladinosyl-3-*O*-carbamoyl Glycosyl Derivatives

**DOI:** 10.3390/molecules22122146

**Published:** 2017-12-04

**Authors:** Chao-Ming Wang, Feng-Lan Zhao, Lei Zhang, Xiao-Yun Chai, Qing-Guo Meng

**Affiliations:** 1 School of Pharmacy, Key Laboratory of Molecular Pharmacology and Drug Evaluation, Ministry of Education, Collaborative Innovation Center of Advanced Drug Delivery System and Biotech Drugs in Universities of Shandong, Yantai University, 30 Qingquan Road, Yantai 264005, China; wangchaoming315@163.com (C.-M.W.); zfl@ytu.edu.cn (F.-L.Z.); fuben19911221@163.com (L.Z.); 2 Department of Organic Chemistry, College of Pharmacy, Second Military Medical University, 325 Guohe Road, Shanghai 200433, China

**Keywords:** azithromycin, glycosyl derivatives, synthesis, antibacterial activity

## Abstract

A novel series of 11,12-cyclic carbonate azithromycin-3-*O*-descladinosyl-3-*O*-carbamoyl glycosyl derivatives were designed, synthesized, and evaluated for their antibacterial activities in vitro. Most of these compounds had significant antibacterial activity against seven kinds of susceptible strains. In particular, compound **G1** exhibited the most potent activity against methicillin-resistant *Streptococcus pneumoniae* 943 (MIC: 1 μg/mL), *Staphylococcus pneumoniae* 746 (MIC: 2 μg/mL), *Streptococcus pyogenes* 447 (MIC: 8 μg/mL), and *Escherichia coli* 236 (MIC: 32 μg/mL), which were two-, four-, four-, four-, and eight-fold stronger activity than azithromycin, respectively. Additionally, compound **G2** exhibited improved activity against methicillin-resistant *Staphylococcus aureus* MRSA-1 (MIC: 8 μg/mL), *Streptococcus pneumoniae* 943 (MIC: 2 μg/mL), *Staphylococcus pneumoniae* 746 (MIC: 2 μg/mL), and *Escherichia coli* 236 (MIC: 32 μg/mL), which were two-, two-, four-, and eight-fold better activity than azithromycin, respectively. As for methicillin-resistant *Staphylococcus aureus* MRSA-1, compound **G6** presented the most excellent activity (MIC: 4 μg/mL), showing four-fold higher activity than azithromycin (MIC: 16 μg/mL) and erythromycin (MIC: 16 μg/mL). However, compared with other compounds, compounds **G7** and **G8** with the disaccharide side chain were observed the lower activity against seven strains.

## 1. Introduction

Macrolide antibiotics are polyketides composed of a 14-, 15-, or 16-membered macrocyclic lactone ring to which several sugars and/or side chains have been attached. They exhibit potent activity against human pathogens in clinical treatment of respiratory tract, genital, and skin infections, inhibiting bacterial protein synthesis by blocking the formation of peptide bond [1,2]. In the past five decades, the broad use of macrolide antibiotics has led to bacterial resistance that can even increase the likelihood of death from infection by common pathogens in developed and developing countries alike [3,4,5]. Therefore, it is still urgently necessary to seek and extend efficacious and safe chemotherapeutic agents to improve present situation. As is known to all, the most widespread approach with combating the resistance is to continue modifying existing classes of antibacterial agents to design and synthesize novel analogues.

**Figure 1:**
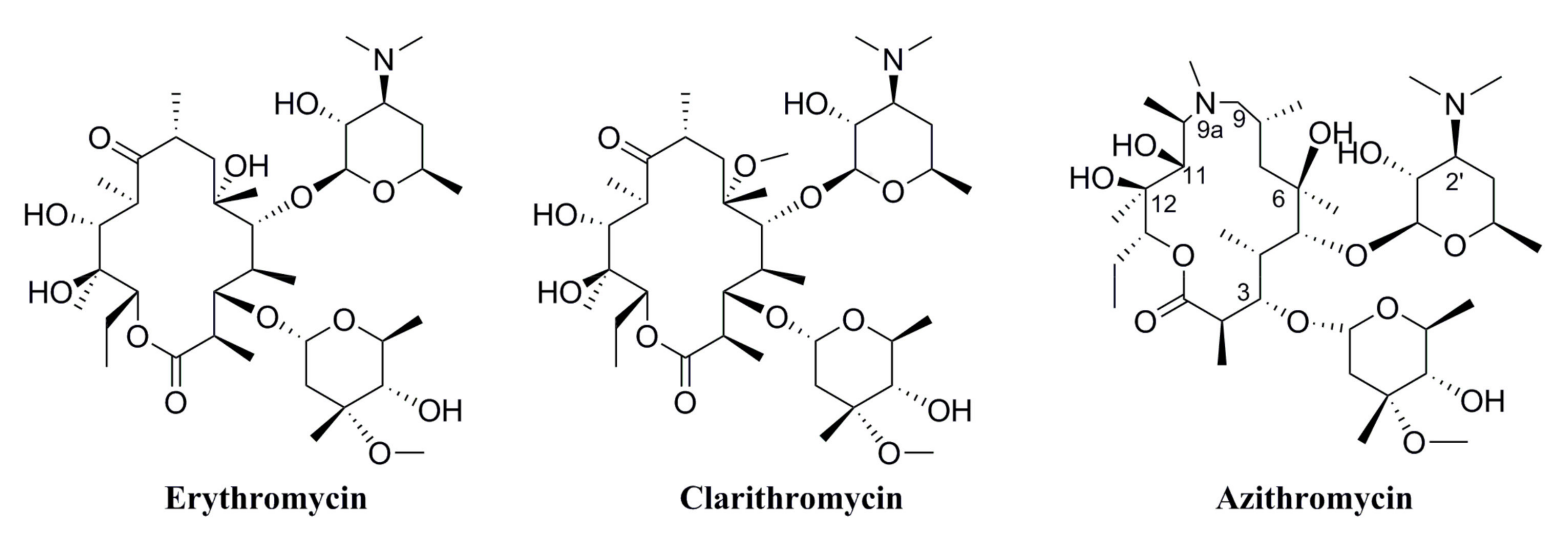
Chemical structure of macrolide antibiotics.

**Figure 2:**
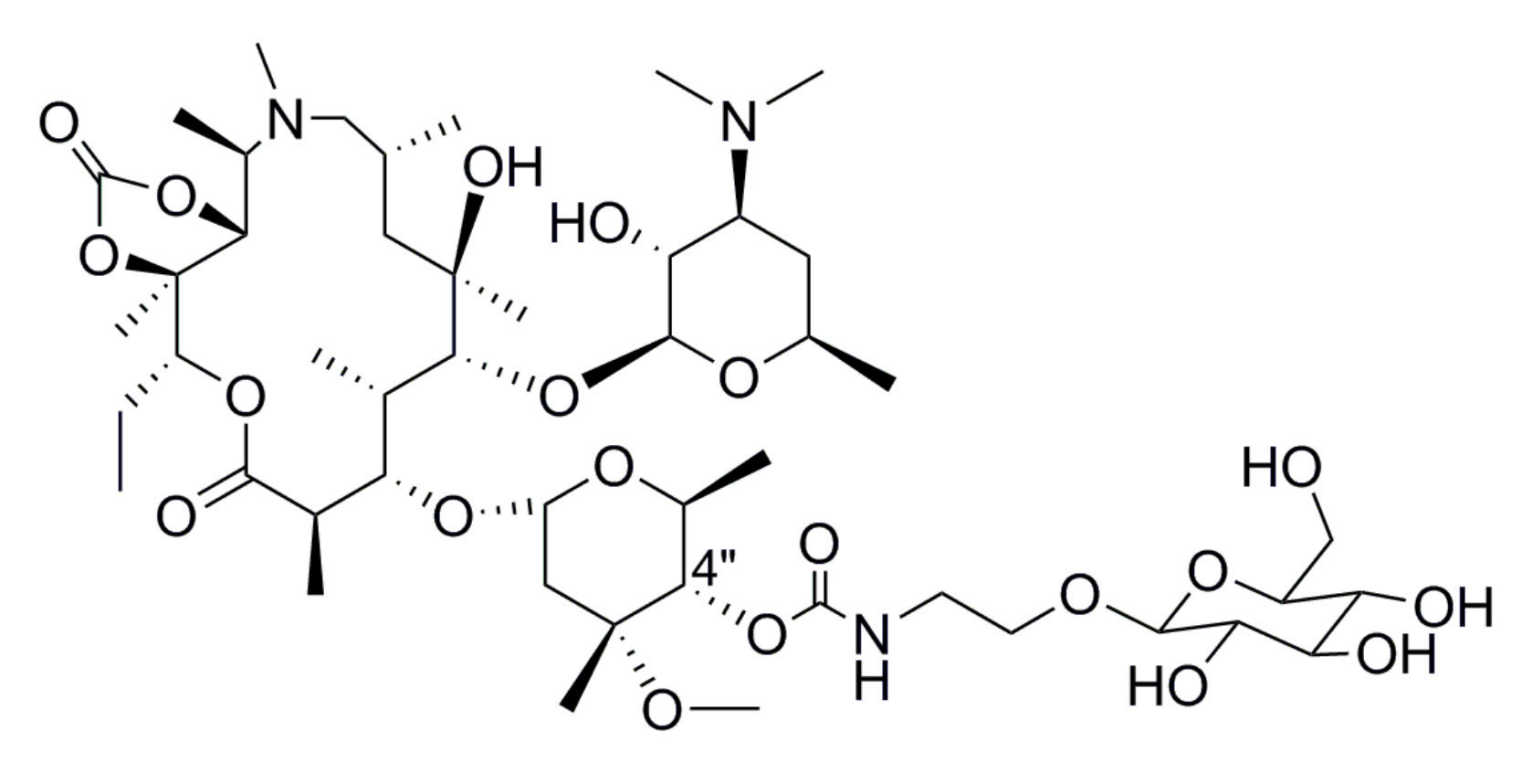
**Figure** **2.** Chemical structure of compound **F1**.

Commonly-used clinical macrolide antibiotics include erythromycin, clarithromycin, and azithromycin (Figure 1). The 14-membered macrolide erythromycin has been widely used for over five decades, but the extensive emergence of erythromycin resistance is extraordinarily high [6]. The 15-membered macrolide azithromycin, which is prepared from erythromycin by expanding semi-synthetically, is characterized by good oral bioavailability, high concentrations in tissues and fluids, wide spectrum of activity, and superior pharmacokinetic and safety properties in comparison to erythromycin [7,8,9,10]. Efforts to find macrolides that were active against macrolide-resistant strains led to the modifications of C-3, C-5, C-6 and C11/C12 positions of the azithromycin [11,12,13]. Additionally, 9a-*N* substituted 15-membered azalides with amide and amine functionalities also had marked advantages over azithromycin in terms of high selectivity for the bacteria and moderate oral bioavailability in vivo [9,14,15,16]. Besides, with intensive research of azithromycin, a novel modified measure of the 4″-position of the cladinose was potent against emergence of drug resistance [17,18,19,20,21]. To resolve these therapeutic problems of tolerance, we investigated and found that the 3-*O*-acylerythromycin derivatives, which were named ‘acylides’, not only recovered the abolished antibacterial activity but also enhanced pharmacological activity against resistant pathogens [22]. There have been few reports about the other glycosylation modification of the 3-*O*-position. Our previous studies have found that the hydroxy group of the glycosyl moiety was significant for hydrogen bond formation, greatly improving soluble in water [19]. Compound **F1**, the 4″-position of the cladinose modified with glucosyl group, was the best active glycosylated compound we reported (Figure 2). It showed improved activity against all the tested bacterial strains relative to the positive control erythromycin, even showed the same or higher antibacterial activities as azithromycin. Besides, we also found that compounds with disaccharide side chains expressed the lowest activity. We deduced that the monosaccharide side chain may help the target compounds fit into the binding pocket and interact with the bacterial ribosome, while the bulky disaccharide side chain might hinder the interactions of the derivative with the bacterial ribosome. Therefore, the group which connected to 3-*O*-position was very important for the activity. Considering these aspects, we hydrolyzed the cladinose of azithromycin and introduced different glycosyl moieties to the 3-*O*-position to examine the antibacterial activities of the derivatives in vitro and discussed the structure–activity relationship.

## 2. Results and Discussions

### 2.1. Chemistry

**Figure 3:**
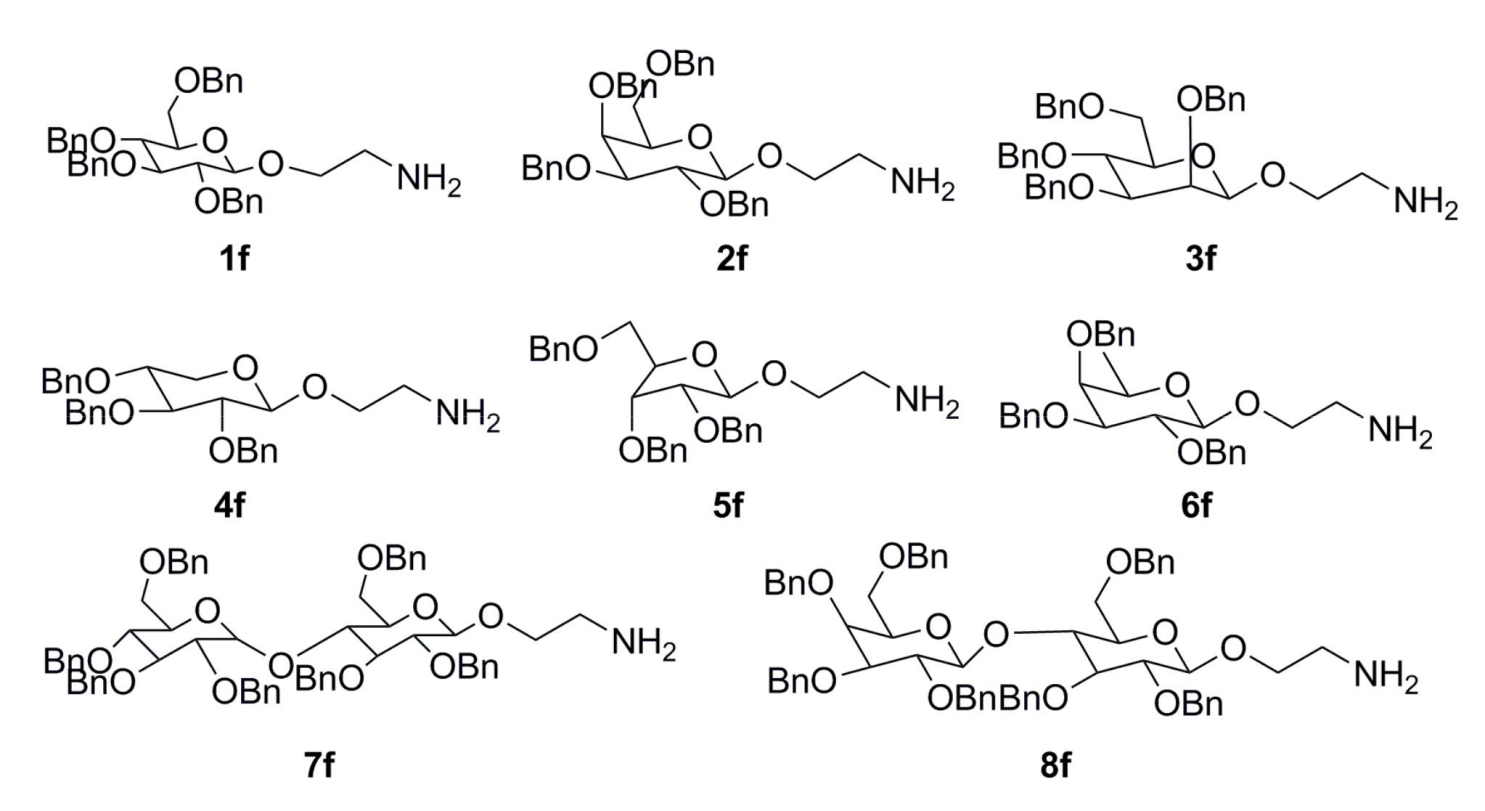
Structure of the intermediates **1f**–**8f**.

**Scheme 1:**
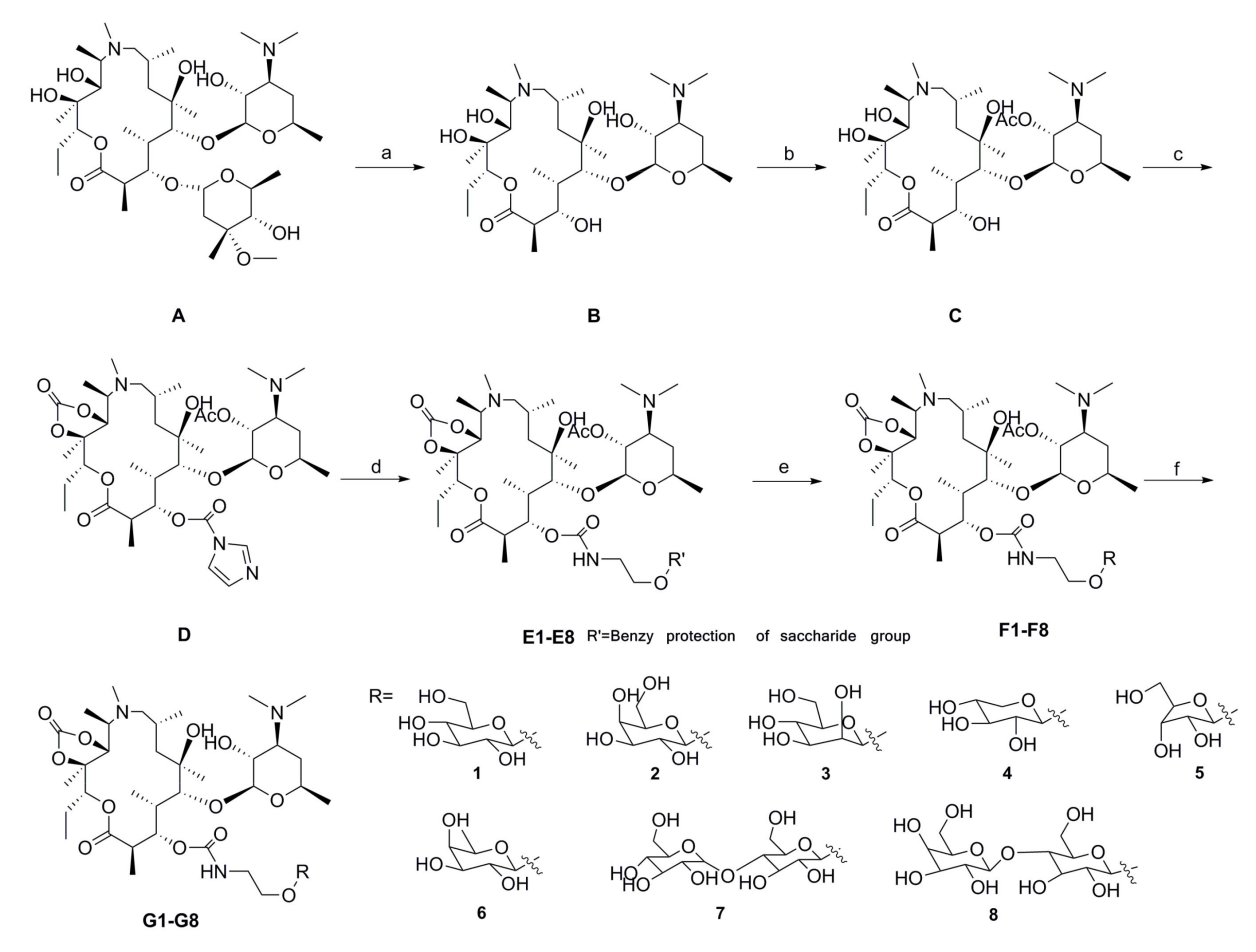
**Scheme** **1.** The synthesis of the target compounds **G1**–**G8**. Reactions and conditions: (**a**) HCl/H_2_O, r.t., 5 h, in 91% yield; (**b**) Ac_2_O, DCM, Et_3_N, r.t., 20 h, in 88% yield; (**c**) CDI, Et_3_N, toluene, reflux, 48 h, in 93% yield; (**d**) **1f**–**8f**, DBU, DMF, r.t., 24 h, in 17–25% yield; (**e**) Pd/C, H_2_, MeOH, r.t., in 70–80% yield; (**f**) CH_3_OH, 55 °C, 20 h, 62–75% yield.

The eight glycosylated intermediate compounds **1f**–**8f** were prepared from inexpensive and available carbohydrates with a series of reported synthetic methods (Figure 3) [19]. The general synthetic route of the target compounds **G1**–**G8** was shown in Scheme 1. First, to remove the l-cladinose, azithromycin was stirred with aqueous HCl and compound **B** was obtained. Then, the 2′-hydroxyl group of compound **B** was protected with acetic anhydride to obtain compound **C**. 11,12-Cyclic carbonate azithromycin 3-*O*-acylimidazolide **D** was prepared by treatment of **C** with Et_3_N and CDI in toluene [23]. NaH and DMF were abandoned in this reaction, greatly improving yield in contrast to previous studies [19]. The compounds **E1**–**E8** were obtained by benzyl protection of the saccharide group in the presence of DBU. The intermediates, **E1**–**E8***,* were converted to compounds **F1**–**F8** by Pd/CaCO_3_ under H_2_. Finally, the target compounds **G1**–**G8** were prepared by deprotection of the 2′-*O*-acetyl group. Structures of all the objective compounds and intermediates were determined by ^1^H-NMR, ^13^C-NMR, and ESI-MS.

### 2.2. Antibacterial Activity

The in vitro antibacterial activities were reported as minimum inhibitory concentrations (MICs), which were determined using a standard dilution assay as recommended by the CLSI [24].

### 2.3. Biological Evaluation

The results of the assays are summarized in Table 1. The data points express the mean of replicate experiments. All of our susceptibility tests were performed three times using each antibacterial agent.

The antibacterial activities indicated that 11,12-cyclic carbonate azithromycin-3-*O*-descladinosyl-3-*O*-carbamoyl glycosyl derivatives exhibited apparent activity against seven strains compared with azithromycin and erythromycin. Noticeably, all the title compounds exhibited more potent against *S. aureus* and *S. pneumoniae* than the other strains. The MIC value indicated that the compounds **G1** and **G2** showed excellent activity against the other compounds, even more potent than the positive control erythromycin and azithromycin. The MIC value of compound **G1** and **G2** (2 µg/mL) was eight times lower than that of the positive control erythromycin (16 µg/mL) against *S. aureus* MSSA-1 in vitro and even the same as the positive control azithromycin (2 µg/mL). In particular, compound **G1** exhibited the most potent activity against methicillin-resistant *S. pneumoniae* 943 (1 μg/mL), *S. pneumoniae* 746 (2 μg/mL), *S. pyogenes* 447 (8 μg/mL), and *E. coli* 236 (32 μg/mL), which were two-, four-, four-, four-, and eight-fold better activity than azithromycin, respectively. Additionally, compound **G2** exhibited improved activity against methicillin-resistant *S. aureus* MRSA-1 (8 μg/mL), *S. pneumoniae* 943 (2 μg/mL), *S. pneumoniae* 746 (2 μg/mL), and *E. coli* 236 (32 μg/mL), which were two-, two-, four-, and eight-fold better activity than azithromycin, respectively. The compounds **G3**–**G6** showed slightly higher activities than the positive control erythromycin and azithromycin, while compounds **G7** and **G8** presented the slightly less activities according to the MIC values. As for methicillin-resistant *S. aureus* MRSA-1, compound **G6** presented the most excellent activity (4 µg/mL), showing four- and four-fold higher activity than azithromycin (16 μg/mL) and erythromycin (16 μg/mL). However, compared with other compounds, compounds **G7** and **G8** with the disaccharide side chain were observed the lower activity against seven strains. Activity of the tested compounds was either maintained or slightly improved against *E. coli* 236 and *E. coli ATCC* 25922. Unfortunately, all compounds exhibited similar or only slightly better activity than erythromycin and azithromycin against *S. pyogenes* 447, *E. coli* 236, and *E. coli ATCC* 25922.

Compared with the 11,12-cyclic carbonate azithromycin-4″-*O*-carbamoyl glycosyl derivatives we reported in the past [19], the title compounds displayed more potent activity against *S. aureus* MSSA-1 and *S. aureus* MRSA-1. Most of them exhibited 2–4 fold higher activity than that before. As for the other five strains, this series did not show notably improved antibacterial activity.

## 3. Materials and Methods

### 3.1. Chemistry

All reactions were monitored by thin-layer chromatography (Huanghai, Yantai, China). A part of each compound was visualized by UV light (Yukang, Shanghai, China). NMR spectra were recorded on a Bruker Avance II 600 spectrometer at 600 MHz for ^1^H-NMR and 150 MHz for ^13^C-NMR with TMS as the internal standard. Chemical shifts are expressed in δ (ppm). The HPLC-MS were recorded on Agilent 1100 series LC/MS. Column chromatography was carried out using silica gel (300–400 mesh, Huanghai, Yantai, China). Silica gel chromatography solvents were of analytical grade (Huanghai, Yantai, China). All reaction solvents were dried prior to use according to standard procedures (Taitan, Shanghai, China).

#### 3.1.1. 3-*O*-descladinosyl Azithromycin (**B**)

We added 36% aqueous HCl dropwise to a solution of azithromycin (10 g, 13 mmol) in water (60 mL) at room temperature until the reaction mixture was adjusted to pH = 1.0–2.0. The resulting solution was allowed to stir for 5 h at the same temperature, and then it was adjusted to pH = 9 with 25% aqueous NH_3_. The precipitate was collected by filtration, and washed with cold water, crystallized from acetone to afford 7.17 g (91%) of 3-*O*-descladinosyazithromycin (**B**) as white solid, M.p. 139–141 °C.

#### 3.1.2. 2′-*O*-Acetyl-3-*O*-descladinosyl Azithromycin (**C**)

Ac_2_O (2.3 mL, 49 mmol) and Et_3_N (6.7 mL, 49 mmol) were added to a solution of 3-*O*-descladinosylazithromycin (7.17 g, 12 mmol) prepared above in CH_2_Cl_2_ (80 mL) at room temperature. The resulting solution was allowed to stir for 24 h at the same temperature. The reaction was quenched with saturated NaHCO_3_ (60 mL) and the aqueous layer was extracted with CH_2_Cl_2_ (2 × 10 mL). The combined organic layers were washed with water and brine, then dried over anhydrous Na_2_SO_4_. The organic layer was filtered and concentrated in vacuum to afford 6.77 g (88%) of 2′-*O*-acetyl-3-*O*-descladinosy azithromycin (**C**) as white solid, M.p. 143–145 °C.

#### 3.1.3. 2′-*O*-Acetyl-3-*O*-acylimidazolyl-3-*O*-descladinosyl Azithromycin-11,12-Cyclic Carbonate (**D**)

Et_3_N (2.2 mL, 14 mmol) and CDI (2.27 g, 14 mmol) were added to a solution of 2′-*O*-acetyl-3-descladinosy azithromycin (**C**, 3.59 g, 5.6 mmol) in toluene (30 mL) . The resulting solution was stirred for 48 h at 80 °C.The reaction was quenched with saturated NaHCO_3_ (30 mL) and the aqueous layer was extracted with toluene (2 × 15 mL). The combined organic layers were dried over anhydrous Na_2_SO_4_, filtered, and concentrated in a vacuum to afford 4.21 g (93%) of compound **D** as white solid, M.p. 149–151 °C.

#### 3.1.4. 2′-*O*-Acetyl-3-*O*-amido-[ethyl-2-(2,3,4,6-tetra-*O*-benzyl-β-d-glucopyranoside)]-3-*O*-descladinosyl Azithromycin-11,12-Cyclic Carbonate (**E1**)

DBU (0.15 mL, 1.03 mmol) and **1f** (0.47 g, 0.79 mmol) were added to a solution of **D** (0.4 g, 0.53 mmol) in DMF (15 mL). The resulting solution was stirred for 24 h at the room temperature. The reaction was quenched with saturated NaHCO_3_ (30 mL) and the aqueous layer was extracted with ethyl acetate (2 × 15 mL). The combined organic layers were washed with water and brine, and dried over NaSO_4_. After filtering and concentrating the reaction solution in vacuum to afford the crude compound **E1** (0.21 g) as white solid. The syntheses of other intermediates (**E2**–**E8**) are carried out according to the above conditions.

#### 3.1.5. 2′-*O*-Acetyl-3-*O*-amido-(ethyl-2-β-d-glucopyranoside)-3-*O*-descladinosyl Azithromycin-11,12-Cyclic Carbonate (**F1**)

A solution of **E1** (140 mg, 0.11 mmol) Pd/C (50 mg) in methanol (15 mL) was adjusted to pH = 4–5 by acetic acid and stirred for 24 h under an atmosphere of hydrogen at the room temperature. After, filtering and concentrating the reaction solution in vacuum afforded the crude compound **F1** as white solid. The syntheses of other intermediates (**F2**–**F8**) are carried out according to the above conditions.

#### 3.1.6. General Procedure for the Preparation of Azithromycin Derivatives **G1**–**G8**

A solution of the above crude intermediate product in MeOH (15 mL) was heated to 55 °C and stirred for 36 h at the same temperature. After concentrating the reaction solution in vacuum, the residue was purified by flash chromatography (DCM/MeOH, 4:1, *v*/*v*) to afford the title compound.

*3-O-Amido-(ethyl-2-β-d-glucopyranoside)-3-O-descladinosyl azithromycin-11,12-cyclic carbonate* (**G1**): White solid; Yield: 75%, M.p. 108–110 °C; ^1^H-NMR (600 MHz, CDCl_3_) δ 5.17 (1H, s), 5.05–5.04 (1H, m), 4.54 (2H, m), 4.38–4.36 (1H, m), 4.26–4.19 (2H, m), 3.99–3.97 (1H, m), 3.87 (1H, t), 3.82 H, m), 3.72–3.70 (1H, m), 3.68 (1H, d, *J* = 1.8 Hz), 3.66 (1H, d, *J* = 5.4 Hz) 3.64–3.61 (1H, m), 3.60 (1H, m), 3.57–3.55 (2H, m), 3.36 (2H, m), 3.27–3.23 (1H, m), 2.96 (1H, m), 2.93 (2H, m), 2.88–2.82 (2H, m), 2.67–2.62 (1H, m), 2.34 (3H, s), 2.52–2.49 (2H, m), 2.25 (9H, m), 1.98 (3H, m), 1.92–1.86 (6H, m), 1.31–1.26 (5H, m), 1.25–1.23 (7H, m), 0.94–0.90 (9H, m); ^13^C-NMR (150 MHz, CDCl_3_) δ 176.7, 170.1, 153.7, l03.6, 100.2, 95.6, 85.9, 79.7, 76.8, 76.7, 76.2, 74.7, 73.4, 73.5, 70.8, 70.3, 68.8, 67.4, 65.3, 63.2, 61.4, 61.1, 50.4, 45.5, 44.2, 41.6, 40.4, 35.7, 34.2, 29.4, 26.7, 22.9, 22.1, 21.2, 15.1, l4.3, 12.2, 10.4, 5.7; MS (ESI) calcd. for C_42_H_73_N_3_O_18_ 907.49; found [M + H]^+^ 908.51. 

*3-O-Amido-(ethyl-2-galactosyl)-3-O-descladinosyl azithromycin-11,12-cyclic carbonate* (**G2**): White solid; Yield: 68%, M.p. 113–115 °C; ^1^H-NMR (600 MHz, CDCl_3_) δ 5.40 (1H, d, *J* = 3.6 Hz), 5.11 (1H, m), 5.02 (1H, d, *J* = 10.5 Hz), 4.50–4.39 (2H, m), 4.34–4.21 (2H, m), 4.33–4.21 (2H, m), 4.18 (1H, d, *J* = 11.4 Hz), 4.12 (1H, m), 4.03 (1H, m), 3.91 (2H, m), 3.70 (2H, m), 3.68–3.54 (2H, m), 3.30 (2H, m), 3.27–3.22 (1H, m), 2.88–2.82 (1H, m), 2.69–2.60 (2H, m), 2.53–2.43 (1H, m), 2.37 (2H, m), 2.25 (9H, m), 2.21 (3H, s), 2.19 (1H, m), 1.91–1.85 (2H, m), 1.68–1.63 (4H, m), 1.45 (3H, m), 1.31–1.23 (4H, m), 1.06–1.01 (9H, m), 0.94–0.90 (9H, m); ^13^C-NMR (150 MHz, CDCl_3_) δ 178.5, 171.1, 153.7, l03.2, 100.7, 94.9, 83.3, 78.4, 77.3, 76.8, 74.7, 74.2, 73.9, 73.7, 73.2, 71.1, 70.3, 70.1, 68.1, 65.5, 62.4, 61.7, 51.2, 45.3, 42.4, 42.1, 40.2, 36.1, 35.2, 29.1, 27.5, 26.8, 22.1, 21.1, 16.4, 14.7, 11.3, 9.2, 5.4; MS (ESI) calcd. for C_42_H_73_N_3_O_18_ 907.49; found [M + H]^+^ 908.51.

*3-O-Amido-(ethyl-2-mannosyl)-3-O-descladinosyl azithromycin-11,12-cyclic carbonate* (**G3**): White solid; Yield: 64%, M.p. 114–116 °C; ^1^H-NMR (600 MHz, CDCl_3_) δ 5.35 (1H, m), 5.04 (1H, m), 4.88 (1H, d, *J* = 3.0 Hz), 4.60 (1H, s), 4.55 (1H, m), 4.40 (1H, d, *J* = 4.2 Hz), 4.30 (1H, d, *J* = 12 Hz), 4.13 (1H, m), 4.04 (1H, m), 3.95 (1H, m), 3.88 (2H, m), 3.82–3.80 (1H, m), 3.68 (2H, m), 3.66 (2H, m), 3.56 (1H, m), 3.47 (2H, m), 3.36–3.35 (2H, m), 3.00–2.95 (3H, m), 2.83 (4H, m), 2.68–2.60 (1H, m), 2.50–2.47 (1H, m), 2.34 (3H, s), 2.25 (6H, m), 2.19 (2H, m), 1.89 (2H, m), 1.66 (2H, m), 1.45–1.42 (5H, m), 1.23 (1H, m), 1.07–1.00 (12H, m), 0.94–0.89 (9H, m); ^13^C-NMR (150 MHz, CDCl_3_) δ 177.6, 171.4, 157.6, 102.3, 101.6, 95.1, 83.7, 78.4, 76.5, 74.4, 74.1, 73.9, 73.2, 70.8, 70.1, 67.8, 65.5, 62.6, 62.1, 50.3, 45.2, 42.1, 40.3, 36.1, 35.1, 29.7, 27.3, 26.6, 21.9, 21.4, 21.2, 20.8, l6.2, l4.4, 11.5, 9.4, 5.3. MS (ESI) calcd. for C_42_H_73_N_3_O_18_ 907.49; found [M + H]^+^ 908.51.

*3-O-Amido-(ethyl-2-ribosyl)-3-O-descladinosyl azithromycin-11,12-cyclic carbonate* (**G4**): White solid; Yield: 71%, M.p. 105–107 °C; ^1^H-NMR (600 MHz, CDCl_3_) δ 5.16 (1H, d, *J* = 4.8 Hz), 5.05–5.04 (1H, m), 4.54 (2H, m), 4.38–4.36 (1H, m), 4.26–4.19 (2H, m), 3.99–3.97 (1H, m), 3.72–3.70 (1H, m), 3.64–3.61 (1H, m), 3.59 (2H, m), 3.57–3.55 (2H, m), 3.53 (1H, d, *J* = 1.8 Hz), 3.51 (1H, d, *J* = 5.4 Hz), 3.47 (1H, m), 3.36 (2H, m), 3.27–3.23 (1H, m), 2.96 (1H, m), 2.91 (2H, m), 2.88–2.82 (2H, m), 2.67–2.62 (1H, m), 2.52–2.49 (2H, m), 2.25 (9H, m), 1.98 (3H, m), 1.92–1.86 (6H, m), 1.31–1.26 (5H, m), 1.25–1.23 (7H, m), 0.94–0.90 (9H, m); ^13^C-NMR (150 MHz, CDCl_3_) δ 177.1, 153.4, 153.2, 103.5, 100.4, 95.6, 85.7, 79.6, 76.7, 76.4, 76.1, 75.1, 74.2, 73.6, 70.6, 68.2, 67.4, 64.8, 62.4, 61.1, 51.4, 45.3, 43.4, 40.0, 35.3, 34.3, 30.l, 26.7, 23.2, 22.0, 21.2, 15.1, 14.4, 11.3, 10.4, 5.8. MS (ESI) calcd.for C_39_H_69_N_3_O_16_ 835.47; found [M + H]^+^ 836.54.

*3-O-Amido-(ethyl-2-xylosyl)-3-O-descladinosyl azithromycin-11,12-cyclic carbonate* (**G5**): White solid; Yield: 65%, M.p. 107–109 °C; ^1^H-NMR (600 MHz, CDCl_3_) δ 5.21(1H, d, *J* = 4.8 Hz), 5.11 (1H, m), 4.50–4.39 (2H, m), 4.34–4.21 (2H, m), 4.33–4.21 (2H, m), 3.83 (1H, d, *J* = 12 Hz), 3.77 (2H, m), 3.74 (1H, d, *J* = 1.8 Hz), 3.71 (1H, d, *J* = 5.4 Hz), 3.68–3.54 (2H, m), 3.47 (2H, m), 3.27–3.22 (1H, m), 3.15(2H, m), 2.88–2.82 (1H, m), 2.69–2.60 (2H, m), 2.53–2.43 (1H, m), 2.37 (2H, m), 2.25 (9H, m), 2.19 (1H, m), 1.91–1.85 (2H, m), 1.68–1.63 (4H, m), 1.45 (3H, m), 1.31–1.23 (4H, m), 1.06–1.01 (9H, m), 0.94–0.90 (9H, m); ^13^C-NMR (150 MHz, CDCl_3_) 177.7, 153.4, 103.2, 101.2, 95.1, 83.6, 78.5, 75.7, 75.4, 74.4, 74.1, 73.5, 71.2, 70.3, 69.4, 68.3, 65.7, 62.3, 51.1, 45.1, 42.6, 40.4, 36.1, 35.4, 29.2, 27.5, 26.8, 21.9, 21.2, 16.2, 14.7, 11.3, 9.1, 5.4; MS (ESI) calcd. for C_39_H_69_N_3_O_16_ 835.47; found [M + H]^+^ 836.54.

*3-O-Amido-(ethyl-2-rhamnosyl)-3-O-descladinosyl azithromycin-11,12-cyclic carbonate* (**G6**): White solid; Yield: 70%, M.p. 110–112 °C; ^1^H-NMR (600 MHz, CDCl_3_) δ 5.55 (1H, s), 5.03 (1H, m), 4.62–4.60 (2H, m), 4.44 (1H, m), 3.79 (1H, m), 3.65–3.55 (2H, m), 3.57 (2H, m), 3.53 (1H, d, *J* = 1.8 Hz), 3.52–3.44 (2H, m), 3.42 (1H, d, *J* = 5.4 Hz), 3.33 (1H, m), 3.28–3.25 (3H, m), 2.49 (1H, m), 2.39–2.37 (1H, m), 2.32–2.20 (10H, m), 2.18 (3H, m),1.91–1.86 (1H, m), 1.77–1.67 (4H, m), 1.48–1.43 (4H, m), 1.30–1.24 (4H, m), 1.04–1.00 (9H, m), 0.96–0.89 (9H, m); ^13^C-NMR (150 MHz, CDCl_3_) δ 177.2, 171.3, 153.4, 145.5, 133.6, 103.1, 101.3, 95.6, 85.9, 79.6, 76.6, 76.1, 74.7, 73.5, 73.3, 71.2, 68.4, 67.5, 64.8, 61.2, 45.1, 43.4, 42.6, 40.2, 35.3, 34.2, 30.1, 26.8, 22.9, 22.0, 21.2, 16.7, l 5.1, l 4.2, l1.4, 10.3, 5.7; MS (ESI) calcd. for C_42_H_73_N_3_O_17_ 891.49; found [M + H]^+^ 892.46.

*3-O-Amido-(ethyl-2-maltosyl)-3-O-descladinosyl azithromycin-11,12-cyclic carbonate* (**G7**): White solid; Yield: 62%, M.p. 117–119 °C; ^1^H-NMR (600 MHz, CDCl_3_) δ 5.41 (1H, d, *J* = 4.2 Hz), 5.03 (1H, m), 4.85 (2H, m), 4.62–4.60 (2H, m), 4.62 (3H, m), 4.53 (2H, m), 4.44 (1H, m), 3.96 (5H, m), 3.65–3.55 (2H, m), 3.33 (1H, m), 3.30 (2H, m), 3.28–3.25 (3H, m), 2.49 (1H, m), 2.39–2.37 (1H, m), 2.32–2.20 (10H, m), 2.14 (3H, m), 1.91–1.86 (1H, m), 1.77–1.67 (4H, m), 1.48–1.43 (4H, m), 1.30–1.24 (4H, m), 1.04–1.00 (9H, m), 0.96–0.89 (9H, m); ^13^C-NMR (150 MHz, CDCl_3_) δ 177.6, 172.3, 153.4, 103.6, 103.1, 101.7, 95.2, 84.1, 78.4, 76.6, 76.3, 74.5, 74.2, 73.8, 73.9, 71.2, 70.1, 68.1, 65.5, 63.2, 62.4, 62.4, 62.3, 51.5, 45.2, 42.5, 40.3, 36.2, 35.2, 29.1, 27.5, 26.8, 22.1, 21.3, l6.3, 14.8, 11.1, 9.4, 5.2; MS (ESI) calcd. for C_48_H_83_N_3_O_23_ 1069.54; found [M + H]^+^ 1070.51.

*3-O-Amido-(ethyl-2-lactosyl)-3-O-descladinosyl azithromycin-11,12-cyclic carbonate* (**G8**): White solid; Yield: 63%, M.p. 119–121 °C; ^1^H-NMR (600 MHz, CDCl_3_) δ 5.24 (1H, d, *J* = 4.8 Hz), 5.13 (1H, m), 4.53 (1H, m), 4.47–4.45 (1H, m), 4.38 (1H, m), 4.34–4.28 (2H, m), 3.92 (2H, d, *J* = 1.8 Hz), 3.83 (2H, m), 3.71 (2H, d, *J* = 10.4 Hz), 3.66 (4H, m), 3.63 (4H, m), 3.55 (2H, m), 3.51–3.47 (2H, m), 3.27–3.22 (2H, m), 3.18 (2H, m), 2.84–2.82 (1H, m), 2.67–2.63 (1H, m), 2.50–2.45 (1H, m), 2.28–2.20 (7H, m), 2.25–2.23 (6H, m), 2.18 (3H, s), 2.01 (1H, m), 1.89 (1H, m), 1.75 (m, 1H), 1.67–1.63 (2H, m), 1.46 (2H, m), 1.25–1.23 (4H, m), 1.05–1.01 (9H, m), 0.94–0.88 (9H, m); ^13^C-NMR (150 MHz, CDCl_3_) δ 177.3, 170.4, l56.7, l53.2, l03.1, 111.2, 101.5, 95.7, 86.1, 85.2, 80.4, 76.5, 76.3, 76.1, 73.5, 73.1, 70.9, 68.5, 67.6, 65.4, 63.1, 62.2, 61.8, 61.2, 53.5, 45.3, 43.5, 41.8, 40.3, 35.5, 34.3, 29.6, 26.8, 26.2, 22.1, 21.9, 21.2, 20.7, 20.7, 15.1, l4.1, l1.4, l0.2, 5.7; MS (ESI) calcd. for C_48_H_83_N_3_O_23_ 1069.54; found [M + H]^+^ 1070.51.

### 3.2. Antibacterial Activity

The in vitro antibacterial activities were reported as minimum inhibitory concentrations (MICs), which were determined using a standard dilution assay as recommended by the CLSI [24]. For the assays, the title compounds to be tested were dissolved in dimethyl sulfoxide (DMSO), serially diluted in growth medium, inoculated and incubated at 35 °C. The selected strains evaluated were methicillin-susceptible *Staphylococcus aureus* MSSA-1 (*S. aureus* MSSA-1), methicillin-resistant *Staphylococcus aureus* MRSA-1 (*S. aureus* MRSA-1), *Streptococcus pneumoniae* 943 (*S. pneumoniae* 943), *Staphylococcus pneumoniae* 746 (*S. pneumoniae* 746), *Streptococcus pyogenes* 447 (*S. pyogenes* 447), *Escherichia coli* 236 (*E. coli* 236), and *Escherichia coli* ATCC 25922 (*E. coli* ATCC 25922). Erythromycin and azithromycin served as the positive control and were obtained from their respective manufacturers.

## 4. Conclusions

In summary, in our endeavor to develop promising antibacterial molecules against the strains based on the naturally occurring azithromycin, we have designed and synthesized a series of related glycosyl derivatives possessing potent in vitro antibacterial activity. The analysis of the in vitro results demonstrated that 11,12-cyclic carbonate azithromycin-3-*O*-descladinosyl-3-*O*-carbamoyl glycosyl derivatives expressed improved antibacterial activities compared with the parent compound. Above all, the compounds **G1**–**G6** with monosaccharide side chain could be suitable to inhibit bacteria protein synthesis in the bacterial ribosome, while compounds **G7** and **G8** with disaccharide side chain exhibited the least activity, which could be due to the long side chain not binding well to the ribosomal RNA of bacteria. Besides, all the compounds showed significantly potent activity (2–16 μg/mL) against *S. aureus* than 11,12-cyclic carbonate azithromycin-4″-*O*-carbamoyl glycosyl derivatives reported in our published paper [19]. Maybe the hydrolyzation of l-cladinose is beneficial for activity improvement. This discovery, as well as further studies on azithromycin glycosyl derivatives, could develop a potent method to overcome the bacterial strain.

## Figures and Tables

**Table 1:** Antibacterial activities of the title compounds in vitro (MIC, μg/mL).

Compd.	*S. aureus* MSSA-1	*S. aureus* MRSA-1	*S. pneumonia*e 943	*S. pneumonia*e 746	*S. pyogenes* 447	*E. coli* 236	*E. coli* ATCC 25922
**G1**	2	8	1	2	8	32	128
**G2**	2	8	2	2	32	32	128
**G3**	4	8	8	4	32	32	128
**G4**	8	8	4	8	32	64	256
**G5**	4	8	4	16	32	64	128
**G6**	2	4	8	16	64	64	128
**G7**	8	16	32	32	128	128	256
**G8**	16	16	16	16	128	256	256
**Azi**	2	16	4	8	32	256	256
**Ery**	16	16	8	16	32	256	256

Abbreviations: **Azi**, azithromycin; **Ery**, erythromycin.
